# Susceptibility to the common cold virus is associated with day length

**DOI:** 10.1016/j.isci.2022.104789

**Published:** 2022-07-19

**Authors:** Cathy A. Wyse, Ava C. Clarke, Enya A. Nordon, Collette Murtagh, Alexandra A. Keogh, Lorna M. Lopez

**Affiliations:** 1 Kathleen Lonsdale Institute for Human Health Research and the Department of Biology, Maynooth University, Kildare, Ireland

**Keywords:** Chronobiology, virology

## Abstract

Seasonal rhythms are endogenous timing mechanisms that allow animals living at temperate latitudes to synchronize their physiology to the seasons. Human viral respiratory disease is prevalent in the winter at temperate latitudes, but the role of endogenous mechanisms in these recurring annual patterns is unclear. The Common Cold Project is a repository of data describing the experimental viral challenge of 1,337 participants across the seasons of the year. We report a secondary analysis of these data to investigate if susceptibility to the common cold is associated with day length. The majority of the participants (78%) showed signs of infection but only 32% developed clinical signs of disease, and the probability of infection was significantly higher in longer day lengths (summer), but the disease was more likely in short (winter) day lengths. The persistence of winter disease patterns in experimental conditions supports the role of endogenous seasonality in human susceptibility to viral infection.

## Highlights

• Outbreaks of viral respiratory disease recur in winter in the northern hemisphere
• In controlled experiments, common cold infection was more likely in summer
• Infection was more likely to progress to the development of cold in winter
• Innate seasonality of human immunity could affect the prevalence of the common cold

## Graphical abstract

**Figure:**
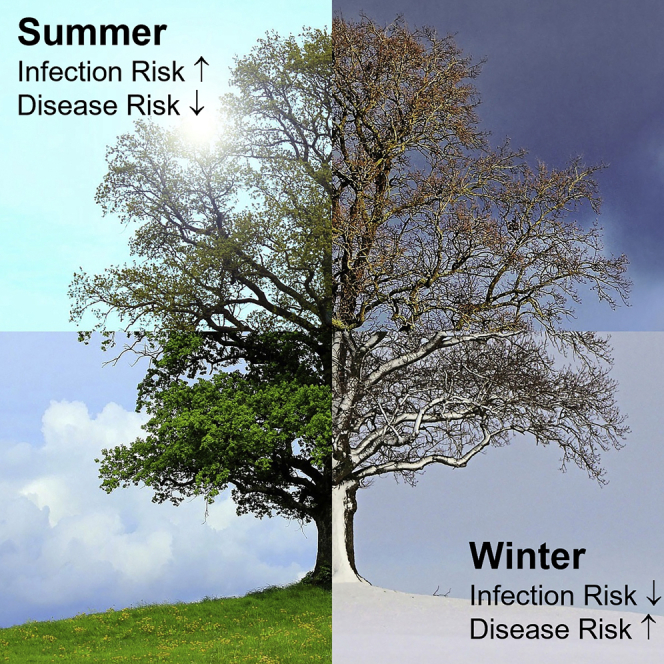

## Introduction

Animals that live at high or low latitudes have evolved annual rhythms in physiology and behavior that can be synchronized with the predictable metabolic, thermoregulatory, and pathogenic challenges presented by the seasons. The mechanisms that mediate this innate seasonality are entrained by day length in temperate Northern and Southern clines, and by seasonal patterns in climatic variables such as rainfall in the tropics, where day length does not vary. (O’Brien, 1993; Wood and Loudon, 2014). Seasonality of reproduction and immunity in temperate regions is driven by day length in animals such as sheep and hamsters (Lincoln, 2019; Onishi et al., 2020), but very little is known about human seasonality, nor how seasonality is regulated in the tropics.

Longitudinal studies of samples submitted to clinical pathology services and surveillance studies across latitudinal clines have shown that respiratory diseases caused by viral infections are prevalent in the winter months in the Northern and Southern hemisphere, and in the rainy season in tropical regions (Baumgartner et al., 2012; Bloom-Feshbach et al., 2013; Morley et al., 2018; Paynter et al., 2015). Furthermore, seasonality is related to latitude within countries; cities in northern regions of India (Chadha et al., 2015) and China (Yu et al., 2013) have winter outbreaks, while southern cities have tropical patterns of respiratory viral disease that track the timing of rainy seasons. The converse is true in Brazil, where winter epidemics occur in the low latitudes of the south, and seasonality gradually attenuates toward central (equatorial) regions (Alonso et al., 2007).

In contrast, respiratory viral infection is not seasonal; viruses are present at all times of the year and the prevalence of infection is not related to clinical disease (Álvarez-Argüelles et al., 2018). The annual prevalence of multiple respiratory viruses (flu, rhinovirus, coronavirus, respiratory syncytial virus) is consistently highest in summer, but its progression to clinical disease is highest in winter (Álvarez-Argüelles et al., 2018; Birger et al., 2018; Galanti et al., 2019; Lee et al., 2012; Shaman et al., 2018). A similar pattern of increased infection in summer and increased disease in winter was also reported for the prevalence of respiratory virus disease in pigs (Poljak et al., 2014). Factors other than the prevalence of infection must mediate winter outbreaks of diseases caused by respiratory viruses and for some reason, infection is unlikely to progress to clinical disease in summer.

In parallel with the entrainment of endogenous seasonality in animals, annual variation in the prevalence of respiratory viral infection is thought to be driven by temperature in temperate regions, and by rainfall patterns and humidity in the tropics (Chan et al., 1999). However, all of these climatic variables are correlated, making their relative contribution as timing cues difficult to decipher. Seasonality is not reliably predicted by any single climatic variable (Baumgartner et al., 2012; Yu et al., 2013) at a population level, and experimental studies have failed to demonstrate any association between temperature and vulnerability to respiratory virus infection under controlled conditions (Dowling et al., 1958; Lofgren et al., 2007). Annual variations in vitamin D (Xu et al., 2016) and human behavior and mobility (Yu et al., 2013) are not strongly associated with seasonal epidemics of winter respiratory viral disease and are unlikely to play more than a contributory role. Seasonal epidemics of respiratory viral infection have challenged healthcare as modern medicine began (Dong, 2011), and their origin remains unclear.

The common cold project is a repository of data from five US and UK viral challenge studies of the factors underlying susceptibility to the common cold. The studies were carried out at temperate latitudes (40-50°N), and across the four seasons, facilitating the investigation of associations between annual changes in day length and susceptibility to experimental viral challenges. If day length-dependent rhythms in human immunity contribute to this susceptibility, then their endogenous origin should cause them to persist when variations in temperature, humidity, and exposure to other sources of infection are eliminated. Here, we test this hypothesis by investigating whether: (i) susceptibility to infection by a respiratory virus or (ii) susceptibility to disease once infection has occurred, are associated with day length following viral challenge under controlled conditions in the Common Cold Project.

## Results

Demographic details of the participants of the PCS and the BCS are shown in Table 1.

**Figure 1:**
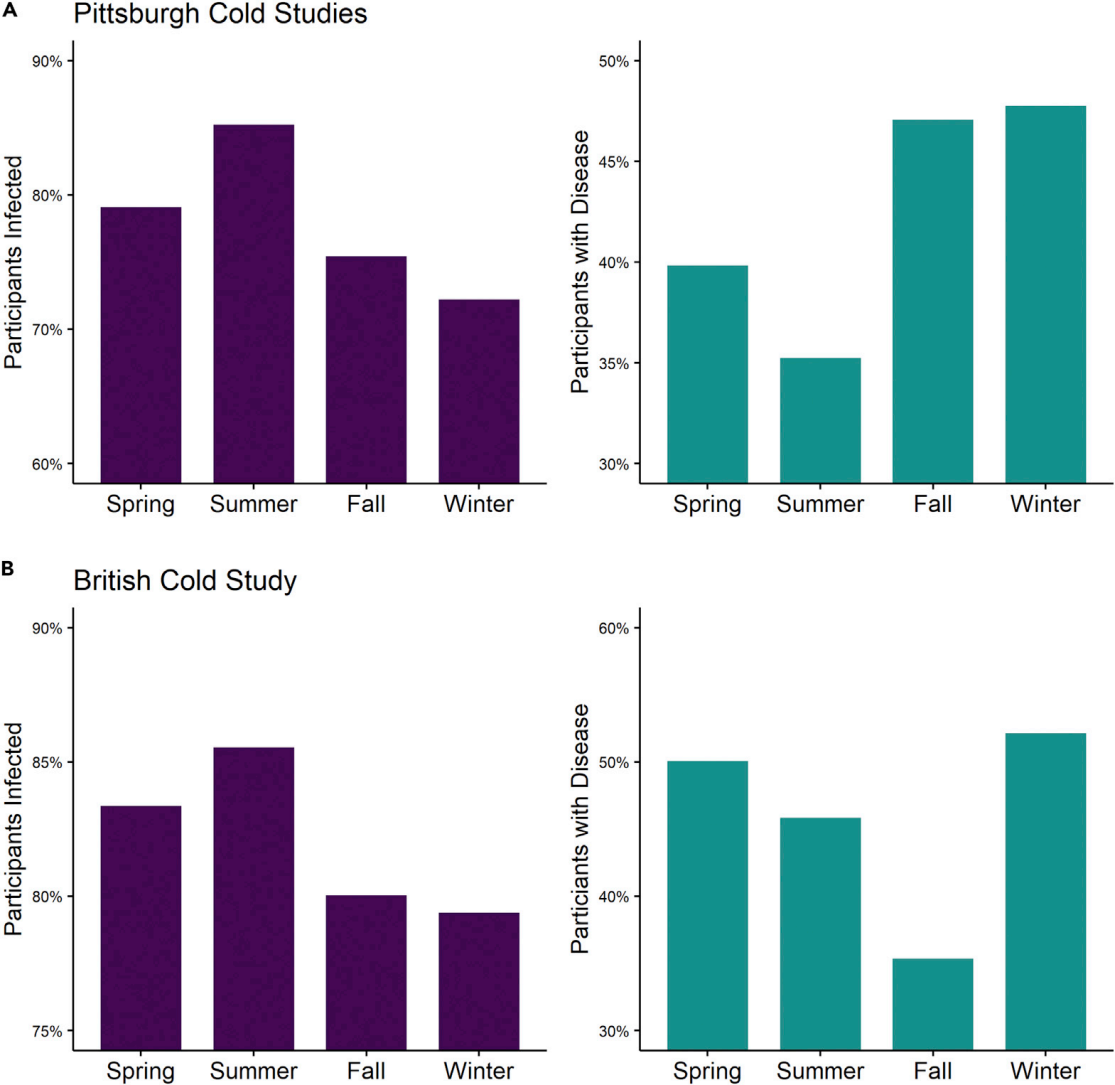
Common cold infection and disease by season (A and B) The percentages of participants infected (A) and that went on to develop clinical signs of disease (B) in the PCS. Infection was higher in summer (June-August) in both studies, and disease was highest in winter (December-February).

**Figure 2:**
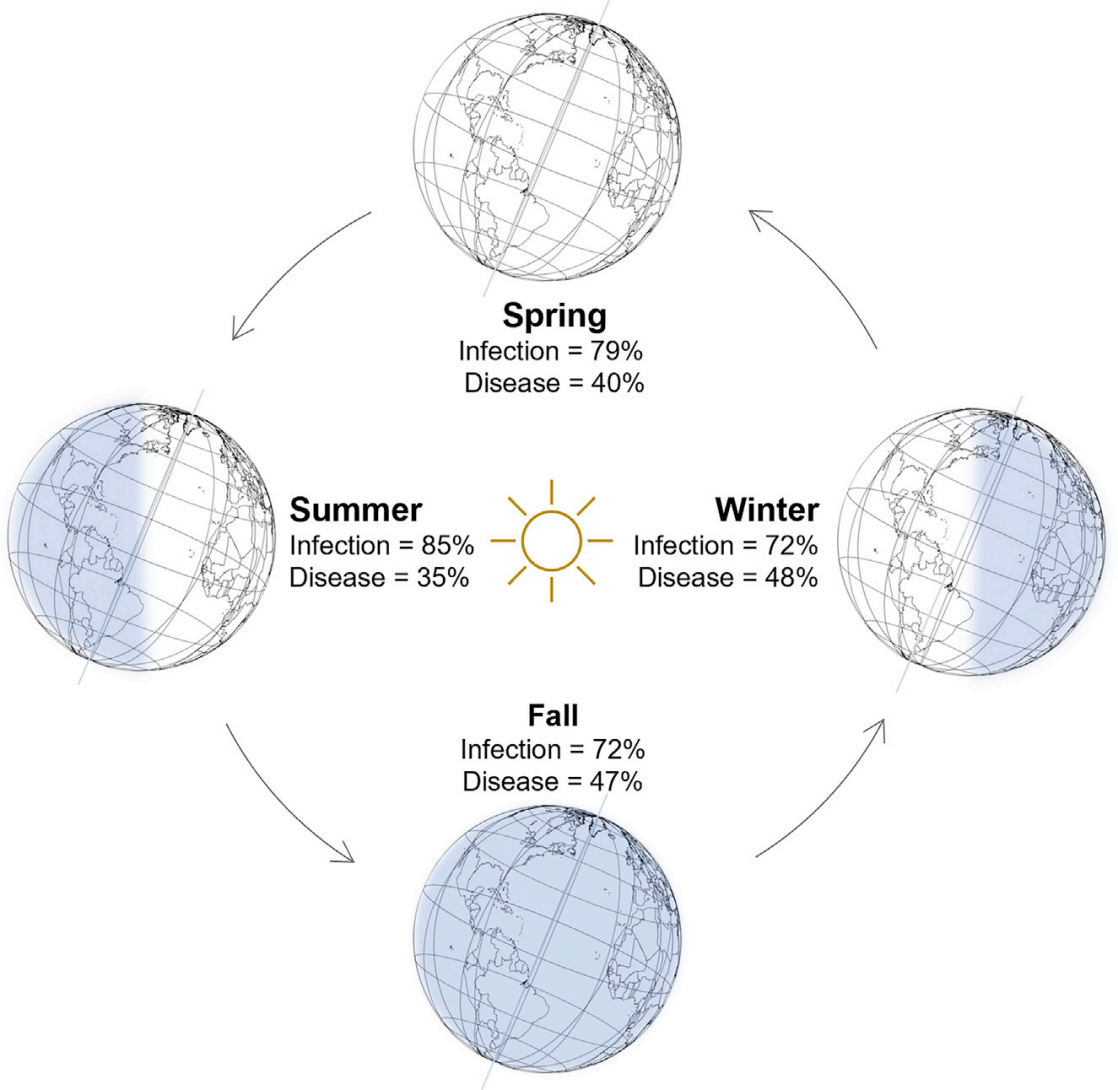
The tilt and orbit of the Earth around the sun generate variation in day length across the year which were associated with variation in susceptibility to common cold infection and disease in this study

Despite the direct application of live virus to the respiratory mucosa, 32% of PCS and 38% of BCS participants did not develop clinical signs of disease after the viral challenge. The majority showed signs of infection (78 and 82% for PCS and BCS), and of those participants that were infected, around half (41 and 46% for PCD and BCS) developed signs of disease. Overall, 22% of the PCS participants and 18% of the BCS evaded infection completely (failed to seroconvert or shed detectable viral particles in nasal secretions), and 58% (PCS) and 54% (BCS) had asymptomatic infection (Tables S1–S4). Univariate analysis directed the selection of parameters (p < 0.2) for inclusion as fixed effects in multivariable models (Table 1). Day length, age, and pre-challenge immunity were significantly associated with the probability of infection post-challenge in the PCS, while day length, pre-challenge immunity, were associated with the probability of developing disease if infected (Table 1). In the PCS, the probability of developing a cold if infected was negatively associated with day length (p < 0.02), suggesting increased susceptibility for infection to progress to disease in winter (Figure 1, Figure 2 and 2). The overall probability of infection was higher in longer day lengths but of borderline statistical significance (Figure 1; Table 1; p = 0.05). Pre-challenge immunity to the challenge virus was strongly associated with both infection (p < 0.001) and the probability of disease given infection (p < 0.001). Older age was associated with increased probability of developing a cold if infected (Table 1; p = 0.01). There were no associations between infection or disease and multiple demographic and lifestyle-associated variables (Tables S1–S4). The BCS followed the same trends for higher probability of infection in longer day lengths, and disease in shorter photoperiods, but these associations did not reach statistical significance (Figure 1).

**Table 1:** Demographic details of the participants in the Pittsburgh Cold Study and the British Cold Study that were included in this study

*Parameter*	Outcome = Infection				Outcome = Disease			
	*Estimate*	*95% CI*		*p*	*Estimate*	*95% CI*		*p*
Daylength	−0.10	−0.18	−0.01	**0.02**	0.08	−0.001	0.16	**0.05**
Pre-challenge immunity	0.40	0.30	0.55	**<0.001**	0.46	0.33	0.64	**<0.001**
Age	0.02	0.01	0.04	**0.01**				
Social Network	−0.01	−0.03	−0.01	0.24				
Sleep Duration	−0.08	−0.18	−0.02	0.11				
Smoking	1.35	0.97	1.89	0.07				
Alcohol					1.36	0.98	1.88	0.07

Estimates are regression coefficients (continuous variables) or odds ratios (categorical and binomial variables) derived from multivariable regression analysis.

**Table 2:** Predictors of infection and disease post viral challenge in the Pittsburgh Cold Study

	Pittsburgh Cold Study (n = 978)	British Cold Study (n = 399)
**Age**		

Mean (SD)	30.51 (±10.34)	33.56 (±2.88)

**Sex**		

Male	485 (50%)	153 (38%)
Female	493 (50%)	246 (62%)

**Body mass index**		

Mean (SD)	26.80 (±6.11)	23.37 (±3.54)

**Pre-challenge immunity**		

No	408 (42%)	192 (48%)
Yes	569 (58%)	132 (33%)
High school or lower	249 (25%)	
College <2 years	297 (30%)	
College >2years + degree	213 (22%)	
Bachelor’s degree or higher	219 (22%)	
O-Levels or lower		228 (57%)
A-Levels		65 (16%)
College, no degree		37 (9%)
Degree or higher		68 (17%)

**Social network**		

Mean (SD)	18.04 (±9.39)	19.1 (±10.24)

**Sleep duration (hours)**		

Mean (SD)	7.04 (±1.534)	7.39 (±1.26)

**Physical activity (PCS)**		

No	227 (23%)	
Yes	750 (77%)	
**Physical activity (BCS, arbitrary units)**		3.64 (±1.5)

**Smoker**		

No	598 (61%)	297 (74%)
Yes	380 (39%)	99 (25%)

**Alcohol (units/week)**		

Mean (SD)	6.24 (±13.59)	13.54 (±16.84)

**Developed infection**		

No	214 (22%)	70 (18%)
Yes	764 (78%)	329 (82%)

**Developed disease**		

No	658 (67%)	248 (62%)
Yes	317 (32%)	151 (38%)

## Discussion

Winter outbreaks of viral respiratory disease recur predictably in all temperate regions on Earth. Annual changes in temperature and humidity are thought to mediate this increased winter prevalence, by increasing survival and transmission of virus in the environment and possibly through changing human behavior. The CCP contains data on the susceptibility of healthy participants to respiratory virus-challenge under controlled, experimental conditions, where the direct impact of the environment on survival and transmission of virus, and on human behavior, are excluded. Here we report that the probability of infection and disease in the CCP were associated with the prevailing photoperiod at the time of viral challenge and seasonal patterns of increased prevalence of colds in winter were retained when viral challenge was presented under experimental conditions. Moreover, the likelihood of infection was higher in long day lengths, confirming previous clinical observations (Lee et al., 2012), and suggesting that the longer days of summer might ameliorate the progression of infection to clinical disease. Participants were quarantined in controlled environments during the viral challenges and the associations we report were independent of confounding lifestyle factors that could affect seasonal vulnerability to seasonal infection, such as habitual physical activity, alcohol intake, smoking, and sleep. The persistence of seasonal patterns of infection and disease in the CCP suggests that factors related to the host immune response might contribute to the winter prevalence of respiratory virus infection.

The physiology and behavior of animals are characterized by timing mechanisms at two fundamental periodicities; ∼24 h circadian “clocks” and ∼12 months circannual “calendars.” These rhythms synchronize biology to the rotational and axial movements of the Earth, optimizing homeostasis by anticipating the variations in light, temperature, and food that these celestial movements generate. Circadian rhythms are generated endogenously by a molecular timing mechanism that is present in virtually every mammalian cell, and that regulates many aspects of human immune function (Cermakian et al., 2022). Seasonal rhythms regulate reproductive, metabolic, behavioral, and immune function in animals at temperate clines, and this action across mammalian systems suggests that circannual timing mechanisms might have a ubiquitous presence in peripheral tissues, analogous to the circadian clock (Lincoln, 2019). Seasonal rhythms are evident in human behavior, metabolism, and immunity (Dopico et al., 2015; Lyall et al., 2018; Wyse et al., 2018), but it is not known if they are a response to climatic seasonal variations or if they represent an endogenous mechanism that would persist in constant conditions.

Viral respiratory disease recurs with seasonality so predictable that annual vaccination programs are routinely planned well in advance of winter outbreaks. Seasonality of respiratory virus infection is strongly dependent on latitude; countries in northern and southern latitudes have outbreaks in their respective wintertime (Bloom-Feshbach et al., 2013), while countries around the equator have outbreaks synchronized with climatic seasons. Latitude is the most consistent predictor of the seasonality of respiratory disease in humans, and its association extends across multiple parameters of seasonality including amplitude, timing, duration, and inter-year variability, supporting a causal relationship (Bloom-Feshbach et al., 2013; Yu et al., 2013). Seasonal changes in day length are a direct function of latitude, and our findings of associations between day length and viral disease are in agreement with previous reports reporting latitude as strongest predictor of the seasonality of respiratory viral diseases (Bloom-Feshbach et al., 2013; Yu et al., 2013).

Outdoor temperature is strongly correlated with day length and we did not attempt to adjust for this potential confounding factor. However, viral challenge was performed under controlled conditions in the Common Cold Study, which eliminates the contribution of factors related to pathogen survival including the sensitivity of the virus to environmental temperature or humidity. The possibility that the participants might be somehow sensitive to the prevailing outdoor temperature remains, but host body temperature does not affect susceptibility to respiratory viral infection (Dowling et al., 1958; Eccles and Wilkinson, 2015). In contrast to the outdoor temperature, there are established mechanisms through which day length regulates immunity in other mammals (Stevenson and Prendergast, 2015) that could also mediate the seasonality of human immune function.

Immune function is seasonal in many animals but the specific changes vary widely between species, and it is likely that seasonal re-programming of immune function serves to optimize the timing of the life cycle of each species to the pathogenic, metabolic and thermoregulatory challenges of their particular environmental niche. Several functional aspects of the human immune system show seasonal patterns (circulating immune cells, acute phase proteins, cytokine signaling, gene expression) (Dopico et al., 2015; Liu and Taioli, 2015; Wyse et al., 2021) and these variations could contribute to our vulnerability to disease in winter. Seasonal rhythms in the human immune system may have evolved to conserve energy during winter when the thermoregulatory and metabolic challenges to survival outweigh those presented by pathogens. The capacity to switch the energetic focus of the immune system from defense to metabolism may have allowed ancient humans to survive the harsh climatic conditions and reduced food availability of winter at northern latitudes. The research field of immunometabolism is revealing evidence for such functional switching of the immune system from defense to metabolism in laboratory animals and at a cellular level (Spiljar et al., 2021), and *in vitro* studies investigating the energetic focus of immune cells across the seasons will progress understanding of the seasonality of human immune function.

The CCP is the largest source of data on human experimental viral challenge to our knowledge, and the sample size of nearly 1,400 participants is strength of our study that enabled the detection of the relatively small effect size of day length on the probability of viral infection and disease. The controlled conditions under which the CCP was performed are also strength, as this eliminated many of the environmental variables that confound studies of natural infections. It is also a strength that we were able to adjust our analysis for multiple demographic and lifestyle variables, which could confound our analysis if they also showed seasonal variations.

### Limitations of the study

The findings of this study are evidence of the seasonality of human susceptibility to common cold viruses, but they must be considered in the context of several important limitations. Firstly, the Common Cold Study data are highly heterogeneous in terms of viral inoculum (rhinovirus, coronavirus, RSV), year of completion (1993-2004), location (US and UK), and pre-existing immunity, and there are many times of the year with missing data. Nevertheless, there was a small effect of seasonality detected in the PCS and a comparable but not statistically significant trend detected in the BCS. It is a limitation that the studies were carried out decades ago, and some aspects may not represent current lifestyle or environmental conditions. However, the seasonal prevalence of the common cold does not vary, and the parameters investigated in this study, day length and vulnerability to seasonal respiratory infection and disease, are likely to be comparable across decades. It is a limitation that the demographic data were self-reported and that ethnic diversity was low and could not be considered in the analysis. It is a strength of this study that data representing a range of day lengths throughout the year were available and that disease induction, diagnosis, and monitoring were quantitative and performed under controlled, quarantined conditions.

The observations of this study provide further evidence that annual rhythms in human immunity might contribute to the seasonality of viral respiratory disease. Seasonal rhythms in human immunity have broad implications for our understanding of the epidemiology of viral infections, and the mechanisms that drive outbreaks of disease. Increased understanding of the role of the host immune system in generating variations in vulnerability to viral respiratory disease could confer novel opportunities for curtailing the spread of novel pathogens, as well as for minimizing the annual impact of circulating viruses such as SARS-CoV-2 and influenza on the health services.

## Institutional permissions

This study was a secondary analysis of de-identified data. The BCS was approved by the Internal Review Board of Carnegie Mellon University and the Harrow District (United Kingdom) Ethical Committee. The remaining studies (PCS1, PCS2, PCS3, PMBC) were approved by both the Carnegie Mellon University and University of Pittsburgh Internal Review Boards.

## STAR★Methods

### Key resources table

**Table:** 

REAGENT or RESOURCE	SOURCE	IDENTIFIER
**Deposited data**		

The Common Cold Project	https://www.cmu.edu/common-cold-project/index.html	Not applicable

**Software and algorithms**		

R	https://www.r-project.org/	R version 4.2.0 (2022-04-22 ucrt)

### Resource availability

#### Lead contact

Further information and requests for resources and reagents should be directed to and will be fulfilled by the lead contact, Cathy Wyse (cathywyse@mu.ie).

#### Materials availability

This study did not generate new unique reagents.

### Experimental model and subject details

Healthy men (n = 638) and women (n = 739) aged 18–55 years were recruited by media advertisement in Pittsburgh (US, 40^o^N) and Salisbury (UK, 51^o^N). The potential influence of sex on outcomes was tested by including sex as a covariable in all analyses. Demographic details of the participants are given in Table 1 and further details of the Common Cold Study are available at www.cmu.edu/common-cold-project/. The BCS and PBS were considered separately due to slight variations in their study design, and because disease outcome was based on self-reported signs in the PCS and clinician-diagnosed in the BCS.

Physical activity was self-reported as the total aerobic exercise per week (BCS), or as a binary variable encoding engagement in physical activity at least once per week (PCS). Social network was the self-reported frequency of close contacts with others, and education was a categorical variable, of no education (1), finished school (2), started college (3), or degree or higher (4). Smoking status was self-reported as current smoker or not. Participants self-reported their number of alcoholic drinks per week, but this parameter was binarized to none (0) or any alcoholic drinks (1) per week because a high proportion of participants did not drink any alcohol. Sleep duration was self-reported in hours per 24h. The date of viral challenge, latitude and longitude were used to derive day length using vectorial algorithms in the ‘*insol’* package in R.

#### Viral challenge

Participants were quarantined and monitored for signs of disease for 2–3 days before and 5–6 days after administration of a culture containing live respiratory virus *(*rhinovirus 2, 9, 14, 39, 21, or 23, respiratory syncytial virus or coronavirus type 229E) directly into the nasal passage (Table S5). There was considerable variability in the strains of virus administered at different months and missing data, particularly in the BCS (Table S5).

In the BCS, disease classification was based on examination by a clinician and scoring across a range of clinical parameters including body temperature, nasal secretion and fatigue. In the PCS, disease status was defined as being infected with the challenge virus and self-reporting signs and symptoms consistent with upper respiratory tract infection (nasal congestion, runny nose, sneezing, cough, sore throat, headache and chills). In both BCS and PCS, infection with common cold virus was defined as showing evidence of viral replication (shedding) in nasal secretions, and/or greater than 4-fold increase in serum specific antibody titre 28 days post challenge. Challenge virus-specific IgG was measured by ELISA 1–2 days prior to viral challenge and 2–60 days post challenge.

### Method details

This study describes a secondary analysis of data collated by the Common Cold Project, which is a repository of data from studies of susceptibility to infection with respiratory viruses carried out in the UK (British Cold Study, BCS) and in the US (Pittsburgh Cold Studies, PCS) between 1986-2004.(“Laboratory for the Study of Stress, Immunity, and Disease. (2016). Common Cold Project. Retrieved from http://www.commoncoldproject.com,” n.d.) The BCS and PCS were analysed separately due to discrepancies between the definition of the disease outcomes and some of the covariables. All study participants gave informed consent; the PCS were approved by the Carnegie Mellon University and University of Pittsburgh Internal Review Boards and the BCS was approved by the Internal Review Board of Carnegie Mellon University and the Harrow District (United Kingdom) Ethical Committee.

### Quantification and statistical analysis

These was extensive variability in the times of year that different viruses were tested in the BCS, and the PCS did not collect data at all in the months of January or February (Table S5). Furthermore, the PCS included data from four separate experiments, Pittsburgh Cold Study 1, Pittsburgh Cold Study 2, Mind-Body Center Study, and Pittsburgh Cold Study 3). We expressed the time-dependent data as a categorical variable (season) or a continuous variable (Day length) rather than month, in order to address the missing data for January and February. Season was defined by the CCP as winter (December-February), spring (March-May), summer (June-August), fall (September-November). Pre-challenge immunity (virus-specific IgG measured at baseline) was defined as positive at antibody titres above 1:2 for rhinoviruses and at antibody titre greater than the sample median for coronavirus or respiratory syncytial virus as previously described for Common Cold Project data (Cohen et al., 2010).

The CCP continuous variables stratified by study name (PCS or BCS) are reported as mean (sd) or n (%) for continuous or categorical data, respectively . The PCS data were not independent due to their derivation from four separate iterations of the PCS. A mixed-effects logistic regression model with PCS as a random effect, was used to address this violation of the assumption of independence. The association between variables and disease outcomes were expressed as odds ratios or regression coefficients with 95% confidence intervals. All analyses were performed using R version 4.1.3 and values of p ≤ 0.05 were considered to represent statistical significance.

## Data Availability

• This paper analyzes existing, publicly available data. • This paper does not report original code. • Any additional information required to reanalyze the data reported in this paper is available from the lead contact upon request.

## References

[bib1] Alonso W.J., Viboud C., Simonsen L., Hirano E.W., Daufenbach L.Z., Miller M.A. (2007). Seasonality of influenza in Brazil: a traveling wave from the amazon to the subtropics. Am. J. Epidemiol..

[bib2] Álvarez-Argüelles M.E., Rojo-Alba S., Pérez Martínez Z., Leal Negredo Á., Boga Riveiro J.A., Alonso Álvarez M.A., Rodríguez Súarez J., de Oña Navarro M., Melón García S. (2018). New clinical and seasonal evidence of infections by Human Parainfluenzavirus. Eur. J. Clin. Microbiol. Infect. Dis..

[bib3] Azziz Baumgartner E., Dao C.N., Nasreen S., Bhuiyan M.U., Mah-E-Muneer S., Mamun A.A., Sharker M.A.Y., Zaman R.U., Cheng P.Y., Klimov A.I. (2012). Seasonality, timing, and climate drivers of influenza activity worldwide. J. Infect. Dis..

[bib4] Birger R., Morita H., Comito D., Filip I., Galanti M., Lane B., Ligon C., Rosenbloom D., Shittu A., Ud-Dean M. (2018). Asymptomatic shedding of respiratory virus among an ambulatory population across seasons. mSphere.

[bib5] Bloom-Feshbach K., Alonso W.J., Charu V., Tamerius J., Simonsen L., Miller M.A., Viboud C. (2013). Latitudinal variations in seasonal activity of influenza and respiratory syncytial virus (RSV): a global comparative Review. PLoS One.

[bib6] Cermakian N., Stegeman S.K., Tekade K., Labrecque N. (2022). Circadian rhythms in adaptive immunity and vaccination. Semin. Immunopathol..

[bib7] Chadha M.S., Potdar V.A., Saha S., Koul P.A., Broor S., Dar L., Chawla-Sarkar M., Biswas D., Gunasekaran P., Abraham A.M. (2015). Dynamics of influenza seasonality at sub-regional levels in India and implications for vaccination timing. PLoS One.

[bib8] Chan P.K.S., Sung R.Y.T., Fung K.S.C., Hui M., Chik K.W., Adeyemi-Doro F.A.B., Cheng A.F. (1999). Epidemiology of respiratory syncytial virus infection among paediatric patients in Hong Kong: seasonality and disease impact. Epidemiol. Infect..

[bib9] Cohen S., Tyrrell D.A.J., Smith A.P. (2010). Psychological stress and susceptibility to the common cold. N. Engl. J. Med..

[bib10] Dong Q. (2011). Seasonal changes and seasonal regimen in hippocrates. J. Cambridge Stud..

[bib11] Dopico X.C., Evangelou M., Ferreira R.C., Guo H., Pekalski M.L., Smyth D.J., Cooper N., Burren O.S., Fulford A.J., Hennig B.J. (2015). Widespread seasonal gene expression reveals annual differences in human immunity and physiology. Nat. Commun..

[bib12] Dowling H.F., Jackson G.G., Spiesman I.G., Inouye T. (1958). Transmission of the common cold to volunteers under controlled conditions. Am. J. Epidemiol..

[bib13] Eccles R., Wilkinson J.E. (2015). Exposure to cold and acute upper respiratory tract infection. Rhinology.

[bib14] Galanti M., Birger R., Ud-Dean M., Filip I., Morita H., Comito D., Anthony S., Freyer G.A., Ibrahim S., Lane B. (2019). Longitudinal active sampling for respiratory viral infections across age groups. Influenza Other Respi. Viruses.

[bib15] Lee W.M., Lemanske R.F., Evans M.D., Vang F., Pappas T., Gangnon R., Jackson D.J., Gern J.E. (2012). Human rhinovirus species and season of infection determine illness severity. Am. J. Respir. Crit. Care Med..

[bib16] Lincoln G. (2019). A brief history of circannual time. J. Neuroendocrinol..

[bib17] Liu B., Taioli E. (2015). Seasonal variations of complete blood count and inflammatory biomarkers in the US population - analysis of NHANES data. PLoS One.

[bib18] Lofgren E., Fefferman N.H., Naumov Y.N., Gorski J., Naumova E.N. (2007). Influenza seasonality: underlying causes and modeling theories. J. Virol..

[bib19] Lyall L.M., Wyse C.A., Celis-Morales C.A., Lyall D.M., Cullen B., Mackay D., Ward J., Graham N., Strawbridge R.J., Gill J.M.R. (2018). Seasonality of depressive symptoms in women but not in men: a cross-sectional study in the UK Biobank cohort. J. Affect. Disord..

[bib20] Morley C., Grimwood K., Maloney S., Ware R.S. (2018). Meteorological factors and respiratory syncytial virus seasonality in subtropical Australia. Epidemiol. Infect..

[bib21] O’Brien G.M. (1993). Seasonal reproduction in flying foxes, reviewed in the context of other tropical mammals. Reprod. Fertil. Dev..

[bib22] Onishi K.G., Maneval A.C., Cable E.C., Tuohy M.C., Scasny A.J., Sterina E., Love J.A., Riggle J.P., Malamut L.K., Mukerji A. (2020). Circadian and circannual timescales interact to generate seasonal changes in immune function. Brain Behav. Immun..

[bib23] Paynter S., Ware R.S., Sly P.D., Williams G., Weinstein P. (2015). Seasonal immune modulation in humans: observed patterns and potential environmental drivers. J. Infect..

[bib24] Poljak Z., Carman S., Mcewen B. (2014). Assessment of seasonality of influenza in swine using field submissions to a diagnostic laboratory in ontario between 2007 and 2012. influenza other respi. Viruses.

[bib25] Shaman J., Morita H., Birger R., Boyle M., Comito D., Lane B., Ligon C., Smith H., Desalle R., Planet P. (2018). Asymptomatic summertime shedding of respiratory viruses. J. Infect. Dis..

[bib26] Spiljar M., Steinbach K., Rigo D., Suárez-Zamorano N., Wagner I., Hadadi N., Vincenti I., Page N., Klimek B., Rochat M.A. (2021). Cold exposure protects from neuroinflammation through immunologic reprogramming. Cell Metab.

[bib27] Stevenson T.J., Prendergast B.J. (2015). Photoperiodic time measurement and seasonal immunological plasticity. Front. Neuroendocrinol..

[bib28] Wood S., Loudon A. (2014). Clocks for all seasons: unwinding the roles and mechanisms of circadian and interval timers in the hypothalamus and pituitary. J. Endocrinol..

[bib29] Wyse C., O’Malley G., Coogan A.N., McConkey S., Smith D.J. (2021). Seasonal and daytime variation in multiple immune parameters in humans: evidence from 329, 261 participants of the UK Biobank cohort. iScience.

[bib30] Wyse C.A., Celis Morales C.A., Ward J., Lyall D., Smith D.J., Mackay D., Curtis A.M., Bailey M.E.S., Biello S., Gill J.M.R., Pell J.P. (2018). Population-level seasonality in cardiovascular mortality, blood pressure, BMI and inflammatory cells in UK biobank. Ann. Med..

[bib31] Xu C., Fang V.J., Perera R.A.P.M., Kam A.M.S., Ng S., Chan Y.H., Chan K.H., Ip D.K.M., Peiris J.M., Cowling B.J. (2016). Serum 25-hydroxyvitamin D was not associated with influenza virus infection in children and adults in Hong Kong, 2009-2010. J. Nutr..

[bib32] Yu H., Alonso W.J., Feng L., Tan Y., Shu Y., Yang W., Viboud C. (2013). Characterization of regional influenza seasonality patterns in China and implications for vaccination strategies: spatio-temporal modeling of surveillance data. PLoS Med..

