# Supplementary material for: Susceptibility to the common cold virus is associated with day length
Source: iScience. 2022 Jul 19;25(8):104789. doi: 10.1016/j.isci.2022.104789 (PMC9379560; doi:10.1016/j.isci.2022.104789)
Supplement: Document S1. Tables S1–S5 [file mmc1.pdf]

iScience, Volume *25*

Supplemental information

Susceptibility to the common cold virus is associated with day length

Cathy A. Wyse, Ava C. Clarke, Enya A. Nordon, Collette Murtagh, Alexandra A. Keogh, and Lorna M. Lopez

Table S1 Demographic details of the participants in the Pittsburgh Cold Study that were infected following experimental challenge with cold virus. Related to Figure 1.

	**No Infection (N=214)**	**Infection (N=764)**	**TOTAL (N=978)**	**p**
**Daylength**				
Mean (SD)	12.05 (± 1.87)	12.35 (± 1.86)	12.29 (± 1.87)	**0.03**
**Age**				
Mean (SD)	29.83 (± 10.19)	30.71 (± 10.38)	30.51 (± 10.34)	0.27
**Sex**				
Male	100 (47 %)	385 (50 %)	485 (50 %)	0.38
Female	114 (53 %)	379 (50 %)	493 (50 %)	
**Body Mass Index**				
Mean (SD)	26.80 (± 6.02)	26.80 (± 6.13)	26.80 (± 6.11)	0.99
**Pre Challenge Immunity**				
No	62 (29 %)	346 (45 %)	408 (42 %)	**<0.001**
Yes	152 (71 %)	417 (55 %)	569 (58 %)	
**Education**				
1	55 (26 %)	194 (25 %)	249 (25 %)	0.89
2	69 (32 %)	228 (30 %)	297 (30 %)	
3	45 (21 %)	168 (22 %)	213 (22 %)	
4	45 (21 %)	174 (23 %)	219 (22 %)	
**Social Network**				
Mean (SD)	18.54 (± 9.44)	17.91 (± 9.37)	18.04 (± 9.39)	0.38
**Sleep Duration (h)**				
Mean (SD)	7.08 (± 1.59)	7.03 (± 1.51)	7.04 (± 1.53)	0.67
**Physical Activity**				
No	49 (23 %)	178 (23 %)	227 (23 %)	0.96
Yes	165 (77 %)	585 (77 %)	750 (77 %)	

	No Infection (N=214)	Infection (N=764)	TOTAL (N=978)	p
**Smoker**				
No	134 (63 %)	464 (61 %)	598 (61 %)	0.67
Yes	80 (37 %)	300 (39 %)	380 (39 %)	
**Alcohol**				
No	142 (66 %)	452 (59 %)	594 (61 %)	0.07
Yes	72 (34 %)	311 (41 %)	383 (39 %)	

Table S2 Demographic details of the participants in the Pittsburgh Cold Study that developed objective signs of cold disease following infection. Related to Figure 1.

	**No Disease (n=444)**	**Developed Disease (n=317)**	**TOTAL (n=764)**	**p**
**Daylength**				
Mean (SD)	12.47 (± 1.84)	12.19 (± 1.82)	12.35 (± 1.86)	**0.04**
**Age**				
Mean (SD)	29.82 (± 10.23)	31.90 (± 10.48)	30.71 (± 10.38)	**0.006**
**Sex**				
Male	228 (51 %)	155 (49 %)	385 (50 %)	0.55
Female	216 (49 %)	162 (51 %)	379 (50 %)	
**Body Mass Index**				
Mean (SD)	26.59 (± 5.56)	27.10 (± 6.87)	26.80 (± 6.13)	0.25
**Pre Challenge Immunity**				
No	167 (38 %)	178 (56 %)	346 (45 %)	**<0.001**
Yes	277 (62 %)	138 (44 %)	417 (55 %)	
**Education**				
1	106 (24 %)	87 (27 %)	194 (25 %)	**0.04**
2	130 (29 %)	97 (31 %)	228 (30 %)	
3	113 (25 %)	54 (17 %)	168 (22 %)	
4	95 (21 %)	79 (25 %)	174 (23 %)	
**Social Network**				
Mean (SD)	18.57 (± 9.56)	17.03 (± 9.06)	17.91 (± 9.37)	**0.02**
**Sleep Duration (h)**				
Mean (SD)	7.11 (± 1.46)	6.92 (± 1.58)	7.03 (± 1.51)	**0.09**
**Physical Activity**				
No	96 (22 %)	81 (26 %)	178 (23 %)	0.22

	No Disease (n=444)	Developed Disease (n=317)	TOTAL (n=764)	p
Yes	348 (78 %)	235 (74 %)	585 (77 %)	
**Smoker**				
No	286 (64 %)	176 (56 %)	464 (61 %)	**0.01**
Yes	158 (36 %)	141 (44 %)	300 (39 %)	
**Alcohol**				
0	262 (59 %)	189 (60 %)	452 (59 %)	0.88
1	182 (41 %)	127 (40 %)	311 (41 %)	

Table S3 Demographic details of the participants in the British Cold Study that were infected following experimental challenge with cold virus. Related to Figure 1.

	**No Infection (n=70)**	**Infection (n=329)**	**TOTAL (n=399)**	**p**
**Daylength**				
Mean (SD)	12.27 (± 2.95)	12.70 (± 2.87)	12.63 (± 2.88)	0.26
**Age**				
Mean (SD)	34.37 (± 11.33)	33.39 (± 10.44)	33.56 (± 10.60)	0.48
**Sex**				
Male	25 (36 %)	128 (39 %)	153 (38 %)	0.71
Female	45 (64 %)	201 (61 %)	246 (62 %)	
**Body Mass Index**				
Mean (SD)	23.65 (± 2.90)	23.31 (± 3.67)	23.37 (± 3.54)	0.46
**Pre Challenge Immunity**				
No	18 (26 %)	174 (53 %)	192 (48 %)	**<0.001**
Yes	38 (54 %)	94 (29 %)	132 (33 %)	
**Physical Activity (BCS, arbitrary units)**				
Mean (SD)	3.31 (± 1.63)	3.71 (± 1.51)	3.64 (± 1.54)	0.35
**Education**				
1	39 (56 %)	189 (57 %)	228 (57 %)	0.41
2	8 (11 %)	57 (17 %)	65 (16 %)	
3	9 (13 %)	28 (9 %)	37 (9 %)	
4	14 (20 %)	54 (16 %)	68 (17 %)	
**Social Network**				
Mean (SD)	19.74 (± 9.50)	18.97 (± 10.40)	19.10 (± 10.24)	0.56
**Smoker**				
No	59 (84 %)	238 (72 %)	297 (74 %)	0.06

	No Infection (n=70)	Infection (n=329)	TOTAL (n=399)	p
Yes	11 (16 %)	88 (27 %)	99 (25 %)	
**Alcohol**				
No	25 (36%)	138 (42%)	163 (41%)	0.40
Yes	45 (64%)	191 (58%)	236 (59%)	
**Sleep Duration**				
Mean (SD)	7.32 (± 1.25)	7.41 (± 1.26)	7.39 (± 1.26)	0.61

Table S4 Demographic details of the participants in the British Cold Study that developed clinical signs of a cold following infection. Related to Figure 1.

	No Disease (n=178)	Developed Disease (n=151)	TOTAL (n=329)	p
**Daylength**				
Mean (SD)	12.86 (± 2.70)	12.52 (± 3.05)	12.70 (± 2.87)	0.27
**Age**				
Mean (SD)	34.22 (± 10.06)	32.40 (± 10.84)	33.39 (± 10.44)	0.11
**Sex**				
Male	75 (42 %)	53 (35 %)	128 (39 %)	0.23
Female	103 (58 %)	98 (65 %)	201 (61 %)	
**Body Mass Index**				
Mean (SD)	23.33 (± 3.62)	23.27 (± 3.736)	23.31 (± 3.67)	0.88
**Pre Challenge Immunity**				
No	79 (44 %)	95 (63 %)	174 (53 %)	**<0.001**
Yes	72 (40 %)	22 (15 %)	94 (29 %)	
**Physical Activity (BCS, arbitrary units)**				
Mean (SD)	3.677 (± 1.51)	3.748 (± 1.51)	3.710 (± 1.51)	0.81
**Education**				
1	99 (56 %)	90 (60 %)	189 (57 %)	0.71
2	30 (17 %)	27 (18 %)	57 (17 %)	
3	15 (8 %)	13 (9 %)	28 (9 %)	
4	33 (19 %)	21 (14 %)	54 (16 %)	
**Social Network**				
Mean (SD)	18.60 (± 10.31)	19.40 (± 10.52)	18.97 (± 10.40)	0.48

	No Disease (n=178)	Developed Disease (n=151)	TOTAL (n=329)	p
**Smoker**				
No	130 (73 %)	108 (72 %)	238 (72 %)	0.66
Yes	45 (25 %)	43 (28 %)	88 (27 %)	
**Alcohol**				
No	64 (36%)	74 (49%)	138 (42%)	**0.02**
Yes	114 (64%)	77 (51%)	191 (58%)	
**Sleep Duration**				
Mean (SD)	7.27 (± 1.17)	7.57 (± 1.35)	7.41 (± 1.29)	**0.03**

Table S5 Viruses used in experimental viral challenges in the PCS and BCS. Data are the number of participants by month for each virus. Shading indicates months where data are available. Related to STAR Methods.

PCS	*Viral Challenge*					BCS	*Viral Challenge*					
Month	Flu	Hanks	rv32	rv39	Total	Month	cov229e	rsv	rv14	rv2	rv9	Total
Jan	0	0	0	0	0	Jan	7	0	20	0	12	39
Feb	0	0	0	0	0	Feb	4	0	24	2	7	37
Mar	9	39	0	100	148	Mar	12	15	0	9	8	44
Apr	0	42	0	63	105	Apr	4	0	0	0	3	7
May	12	0	41	220	273	May	0	15	18	0	0	33
Jun	0	0	0	0	0	Jun	0	20	8	0	9	37
Jul	0	0	0	75	75	Jul	1	3	0	9	36	49
Aug	0	0	0	53	53	Aug	0	0	0	24	28	52
Sep	10	0	35	30	75	Sep	0	0	0	35	19	54
Oct	0	0	0	23	23	Oct	8	2	0	7	4	21
Nov	0	48	0	87	135	Nov	3	0	7	0	0	10
Dec	7	0	30	92	129	Dec	1	0	15	0	0	16
Total	38	129	106	743	1016	***Total***	40	55	92	86	126	399
